# Supplementary material for: Modeling glioblastoma heterogeneity as a dynamic network of cell states
Source: Mol Syst Biol. 2021 Sep 16;17(9):e10105. doi: 10.15252/msb.202010105 (PMC8444284; doi:10.15252/msb.202010105)
Supplement: Supplementary file 5 — Source Data for Figure 3 [file MSB-17-e10105-s001.zip › Figure3A_sourcedata/GSEA_3065/nosigdb_state3.GseaPreranked.1622718426028/RICHARDS_NOWAKOWSKI_SCIENCE_2017_VRG_UPREG.html]

Details for gene set RICHARDS\_NOWAKOWSKI\_SCIENCE\_2017\_VRG\_UPREG[GSEA]

|  || Dataset | state3 |
| Phenotype | NoPhenotypeAvailable |
| Upregulated in class | na\_pos |
| GeneSet | RICHARDS\_NOWAKOWSKI\_SCIENCE\_2017\_VRG\_UPREG |
| Enrichment Score (ES) | 0.3897315 |
| Normalized Enrichment Score (NES) | 1.7753677 |
| Nominal p-value | 0.0 |
| FDR q-value | 0.0071266154 |
| FWER p-Value | 0.078 |
Table: GSEA Results Summary

  

Fig 1: Enrichment plot: RICHARDS\_NOWAKOWSKI\_SCIENCE\_2017\_VRG\_UPREG      
 Profile of the Running ES Score & Positions of GeneSet Members on the Rank Ordered List

  

| PROBE | GENE SYMBOL | GENE\_TITLE | RANK IN GENE LIST | RANK METRIC SCORE | RUNNING ES | CORE ENRICHMENT || 1 | BCAN |  |  | 0 | 0.883 | 0.0174 | Yes |
| 2 | PTPRZ1 |  |  | 14 | 0.532 | 0.0264 | Yes |
| 3 | SYNE2 |  |  | 16 | 0.529 | 0.0367 | Yes |
| 4 | PTN |  |  | 22 | 0.493 | 0.0459 | Yes |
| 5 | CANX |  |  | 27 | 0.479 | 0.0549 | Yes |
| 6 | SQLE |  |  | 39 | 0.457 | 0.0626 | Yes |
| 7 | FGFBP3 |  |  | 51 | 0.431 | 0.0698 | Yes |
| 8 | PON2 |  |  | 63 | 0.389 | 0.0762 | Yes |
| 9 | TMBIM6 |  |  | 73 | 0.372 | 0.0824 | Yes |
| 10 | HMGCR |  |  | 77 | 0.369 | 0.0894 | Yes |
| 11 | FAT1 |  |  | 95 | 0.350 | 0.0942 | Yes |
| 12 | SGCE |  |  | 101 | 0.344 | 0.1004 | Yes |
| 13 | GPM6B |  |  | 105 | 0.343 | 0.1069 | Yes |
| 14 | SCD |  |  | 108 | 0.340 | 0.1133 | Yes |
| 15 | SYT11 |  |  | 111 | 0.335 | 0.1197 | Yes |
| 16 | NES |  |  | 113 | 0.332 | 0.1262 | Yes |
| 17 | SEMA5A |  |  | 121 | 0.324 | 0.1317 | Yes |
| 18 | CALD1 |  |  | 124 | 0.322 | 0.1378 | Yes |
| 19 | EMP2 |  |  | 135 | 0.315 | 0.1429 | Yes |
| 20 | SLC16A1 |  |  | 138 | 0.314 | 0.1488 | Yes |
| 21 | TJP1 |  |  | 139 | 0.314 | 0.1550 | Yes |
| 22 | DAD1 |  |  | 144 | 0.313 | 0.1607 | Yes |
| 23 | TTYH1 |  |  | 183 | 0.293 | 0.1620 | Yes |
| 24 | MYO10 |  |  | 206 | 0.280 | 0.1649 | Yes |
| 25 | DAG1 |  |  | 209 | 0.278 | 0.1702 | Yes |
| 26 | JAM2 |  |  | 217 | 0.275 | 0.1748 | Yes |
| 27 | SEPT7 |  |  | 221 | 0.274 | 0.1798 | Yes |
| 28 | MLEC |  |  | 223 | 0.273 | 0.1851 | Yes |
| 29 | DNAJC1 |  |  | 227 | 0.272 | 0.1901 | Yes |
| 30 | RCN1 |  |  | 233 | 0.270 | 0.1948 | Yes |
| 31 | DHCR24 |  |  | 244 | 0.265 | 0.1989 | Yes |
| 32 | ARL6IP5 |  |  | 245 | 0.265 | 0.2041 | Yes |
| 33 | FKBP9 |  |  | 246 | 0.265 | 0.2094 | Yes |
| 34 | TNC |  |  | 249 | 0.264 | 0.2143 | Yes |
| 35 | HMGCS1 |  |  | 267 | 0.254 | 0.2173 | Yes |
| 36 | PTPRG |  |  | 268 | 0.254 | 0.2223 | Yes |
| 37 | NOTCH1 |  |  | 272 | 0.253 | 0.2270 | Yes |
| 38 | ITM2C |  |  | 273 | 0.252 | 0.2319 | Yes |
| 39 | VCAN |  |  | 293 | 0.245 | 0.2345 | Yes |
| 40 | STAG2 |  |  | 304 | 0.242 | 0.2381 | Yes |
| 41 | SALL3 |  |  | 312 | 0.240 | 0.2420 | Yes |
| 42 | IL6ST |  |  | 316 | 0.239 | 0.2464 | Yes |
| 43 | JAM3 |  |  | 317 | 0.239 | 0.2511 | Yes |
| 44 | ITGAV |  |  | 322 | 0.238 | 0.2553 | Yes |
| 45 | MYO6 |  |  | 363 | 0.227 | 0.2550 | Yes |
| 46 | CTNNB1 |  |  | 373 | 0.224 | 0.2584 | Yes |
| 47 | TMED10 |  |  | 378 | 0.223 | 0.2623 | Yes |
| 48 | SEMA6A |  |  | 387 | 0.222 | 0.2657 | Yes |
| 49 | LPCAT1 |  |  | 404 | 0.218 | 0.2681 | Yes |
| 50 | MSMO1 |  |  | 413 | 0.215 | 0.2714 | Yes |
| 51 | FGFR1 |  |  | 419 | 0.215 | 0.2751 | Yes |
| 52 | ELOVL5 |  |  | 450 | 0.209 | 0.2756 | Yes |
| 53 | AKAP13 |  |  | 452 | 0.209 | 0.2796 | Yes |
| 54 | DDR1 |  |  | 454 | 0.208 | 0.2836 | Yes |
| 55 | LRP6 |  |  | 458 | 0.207 | 0.2874 | Yes |
| 56 | ITGB8 |  |  | 463 | 0.206 | 0.2910 | Yes |
| 57 | MAGI1 |  |  | 476 | 0.202 | 0.2935 | Yes |
| 58 | TFPI |  |  | 480 | 0.202 | 0.2971 | Yes |
| 59 | FKBP10 |  |  | 481 | 0.201 | 0.3011 | Yes |
| 60 | CREB5 |  |  | 497 | 0.199 | 0.3033 | Yes |
| 61 | LDLR |  |  | 498 | 0.199 | 0.3072 | Yes |
| 62 | SLC25A37 |  |  | 500 | 0.199 | 0.3110 | Yes |
| 63 | ITGA6 |  |  | 502 | 0.198 | 0.3148 | Yes |
| 64 | PHIP |  |  | 538 | 0.194 | 0.3144 | Yes |
| 65 | TMEM98 |  |  | 593 | 0.187 | 0.3117 | Yes |
| 66 | APC |  |  | 616 | 0.184 | 0.3127 | Yes |
| 67 | AKAP12 |  |  | 640 | 0.180 | 0.3135 | Yes |
| 68 | ROBO1 |  |  | 644 | 0.179 | 0.3167 | Yes |
| 69 | SPAG9 |  |  | 667 | 0.176 | 0.3176 | Yes |
| 70 | COL11A1 |  |  | 684 | 0.173 | 0.3191 | Yes |
| 71 | NTRK3 |  |  | 687 | 0.173 | 0.3223 | Yes |
| 72 | AASS |  |  | 696 | 0.170 | 0.3247 | Yes |
| 73 | SLC35F1 |  |  | 711 | 0.168 | 0.3263 | Yes |
| 74 | RYK |  |  | 715 | 0.168 | 0.3293 | Yes |
| 75 | SLC1A3 |  |  | 741 | 0.165 | 0.3296 | Yes |
| 76 | CST3 |  |  | 749 | 0.164 | 0.3320 | Yes |
| 77 | SLC12A2 |  |  | 751 | 0.164 | 0.3351 | Yes |
| 78 | UBXN4 |  |  | 753 | 0.163 | 0.3382 | Yes |
| 79 | EZR |  |  | 759 | 0.162 | 0.3408 | Yes |
| 80 | PAG1 |  |  | 761 | 0.162 | 0.3439 | Yes |
| 81 | MEGF10 |  |  | 762 | 0.162 | 0.3471 | Yes |
| 82 | KIF1B |  |  | 767 | 0.161 | 0.3498 | Yes |
| 83 | LRIG1 |  |  | 772 | 0.161 | 0.3525 | Yes |
| 84 | CLDND1 |  |  | 773 | 0.160 | 0.3556 | Yes |
| 85 | SNX13 |  |  | 775 | 0.160 | 0.3587 | Yes |
| 86 | FSTL1 |  |  | 790 | 0.159 | 0.3602 | Yes |
| 87 | RDX |  |  | 797 | 0.158 | 0.3626 | Yes |
| 88 | FAM171B |  |  | 807 | 0.157 | 0.3646 | Yes |
| 89 | PHLPP1 |  |  | 819 | 0.156 | 0.3663 | Yes |
| 90 | VCL |  |  | 834 | 0.154 | 0.3677 | Yes |
| 91 | NAPEPLD |  |  | 847 | 0.153 | 0.3693 | Yes |
| 92 | CYR61 |  |  | 873 | 0.150 | 0.3693 | Yes |
| 93 | LAMA1 |  |  | 930 | 0.145 | 0.3655 | Yes |
| 94 | HSPH1 |  |  | 937 | 0.144 | 0.3676 | Yes |
| 95 | IQGAP2 |  |  | 943 | 0.143 | 0.3698 | Yes |
| 96 | HTRA1 |  |  | 955 | 0.142 | 0.3713 | Yes |
| 97 | ETV1 |  |  | 963 | 0.141 | 0.3733 | Yes |
| 98 | STAT3 |  |  | 965 | 0.141 | 0.3759 | Yes |
| 99 | MSN |  |  | 967 | 0.141 | 0.3786 | Yes |
| 100 | PTGFRN |  |  | 979 | 0.140 | 0.3800 | Yes |
| 101 | CLU |  |  | 988 | 0.139 | 0.3818 | Yes |
| 102 | PTPRF |  |  | 1007 | 0.138 | 0.3824 | Yes |
| 103 | MLC1 |  |  | 1033 | 0.136 | 0.3821 | Yes |
| 104 | RBM39 |  |  | 1043 | 0.135 | 0.3837 | Yes |
| 105 | TMX1 |  |  | 1053 | 0.134 | 0.3853 | Yes |
| 106 | ACTN1 |  |  | 1079 | 0.132 | 0.3849 | Yes |
| 107 | IFI6 |  |  | 1089 | 0.131 | 0.3864 | Yes |
| 108 | MRC2 |  |  | 1162 | 0.125 | 0.3803 | Yes |
| 109 | PTPN13 |  |  | 1167 | 0.124 | 0.3823 | Yes |
| 110 | TMEM132B |  |  | 1194 | 0.122 | 0.3816 | Yes |
| 111 | FZD3 |  |  | 1258 | 0.117 | 0.3764 | Yes |
| 112 | LIFR |  |  | 1260 | 0.117 | 0.3786 | Yes |
| 113 | ZDHHC13 |  |  | 1268 | 0.117 | 0.3800 | Yes |
| 114 | CCND2 |  |  | 1286 | 0.116 | 0.3803 | Yes |
| 115 | TTYH3 |  |  | 1287 | 0.116 | 0.3826 | Yes |
| 116 | SFRP1 |  |  | 1291 | 0.116 | 0.3845 | Yes |
| 117 | SRGAP3 |  |  | 1353 | 0.111 | 0.3794 | Yes |
| 118 | ATXN2 |  |  | 1366 | 0.110 | 0.3802 | Yes |
| 119 | SIPA1L1 |  |  | 1377 | 0.110 | 0.3811 | Yes |
| 120 | CNTLN |  |  | 1378 | 0.110 | 0.3833 | Yes |
| 121 | NPHP3 |  |  | 1416 | 0.107 | 0.3810 | Yes |
| 122 | TSPAN6 |  |  | 1430 | 0.107 | 0.3816 | Yes |
| 123 | INTU |  |  | 1441 | 0.106 | 0.3825 | Yes |
| 124 | GTF2H2 |  |  | 1458 | 0.105 | 0.3826 | Yes |
| 125 | TSPAN5 |  |  | 1466 | 0.105 | 0.3839 | Yes |
| 126 | LIPG |  |  | 1468 | 0.105 | 0.3858 | Yes |
| 127 | PDPN |  |  | 1483 | 0.104 | 0.3862 | Yes |
| 128 | HES1 |  |  | 1501 | 0.103 | 0.3862 | Yes |
| 129 | CNTNAP3B |  |  | 1511 | 0.103 | 0.3872 | Yes |
| 130 | GPC4 |  |  | 1512 | 0.102 | 0.3892 | Yes |
| 131 | DKK3 |  |  | 1538 | 0.101 | 0.3882 | Yes |
| 132 | GAB1 |  |  | 1555 | 0.100 | 0.3883 | Yes |
| 133 | RAB3GAP2 |  |  | 1575 | 0.099 | 0.3880 | Yes |
| 134 | ZBTB20 |  |  | 1632 | 0.095 | 0.3832 | Yes |
| 135 | MSI2 |  |  | 1640 | 0.095 | 0.3842 | Yes |
| 136 | MEIS1 |  |  | 1663 | 0.093 | 0.3834 | Yes |
| 137 | SOAT1 |  |  | 1674 | 0.093 | 0.3841 | Yes |
| 138 | FUT8 |  |  | 1685 | 0.093 | 0.3847 | Yes |
| 139 | LPIN2 |  |  | 1687 | 0.093 | 0.3864 | Yes |
| 140 | C1orf61 |  |  | 1703 | 0.091 | 0.3864 | Yes |
| 141 | OTUD7B |  |  | 1717 | 0.091 | 0.3867 | Yes |
| 142 | LPP |  |  | 1725 | 0.090 | 0.3876 | Yes |
| 143 | SYNE1 |  |  | 1729 | 0.090 | 0.3890 | Yes |
| 144 | CA12 |  |  | 1739 | 0.089 | 0.3897 | Yes |
| 145 | SAMD4A |  |  | 1784 | 0.087 | 0.3862 | No |
| 146 | SCAF11 |  |  | 1822 | 0.085 | 0.3835 | No |
| 147 | AGO3 |  |  | 1854 | 0.084 | 0.3814 | No |
| 148 | PCMTD1 |  |  | 1857 | 0.083 | 0.3828 | No |
| 149 | BMP7 |  |  | 1871 | 0.082 | 0.3829 | No |
| 150 | MLLT10 |  |  | 1959 | 0.078 | 0.3740 | No |
| 151 | PSAP |  |  | 1988 | 0.076 | 0.3722 | No |
| 152 | NOTCH2 |  |  | 1990 | 0.076 | 0.3736 | No |
| 153 | APBB2 |  |  | 2011 | 0.076 | 0.3727 | No |
| 154 | AXL |  |  | 2020 | 0.075 | 0.3732 | No |
| 155 | FOXK1 |  |  | 2028 | 0.075 | 0.3739 | No |
| 156 | ARHGEF40 |  |  | 2118 | 0.071 | 0.3647 | No |
| 157 | ARAP2 |  |  | 2133 | 0.071 | 0.3644 | No |
| 158 | CSMD2 |  |  | 2213 | 0.068 | 0.3563 | No |
| 159 | TRIM9 |  |  | 2215 | 0.068 | 0.3575 | No |
| 160 | LRRC17 |  |  | 2223 | 0.067 | 0.3580 | No |
| 161 | GRB14 |  |  | 2251 | 0.066 | 0.3561 | No |
| 162 | IDI1 |  |  | 2285 | 0.065 | 0.3534 | No |
| 163 | TRIB2 |  |  | 2296 | 0.065 | 0.3535 | No |
| 164 | ID4 |  |  | 2306 | 0.064 | 0.3537 | No |
| 165 | HSPA4L |  |  | 2311 | 0.064 | 0.3545 | No |
| 166 | RALGAPA2 |  |  | 2340 | 0.063 | 0.3524 | No |
| 167 | RFTN2 |  |  | 2346 | 0.063 | 0.3530 | No |
| 168 | NOTCH3 |  |  | 2367 | 0.062 | 0.3519 | No |
| 169 | TLN2 |  |  | 2378 | 0.062 | 0.3519 | No |
| 170 | ADAMTS6 |  |  | 2418 | 0.060 | 0.3484 | No |
| 171 | SHC3 |  |  | 2460 | 0.058 | 0.3447 | No |
| 172 | CUL3 |  |  | 2465 | 0.058 | 0.3454 | No |
| 173 | MEIS2 |  |  | 2506 | 0.057 | 0.3417 | No |
| 174 | ATP2B4 |  |  | 2510 | 0.057 | 0.3425 | No |
| 175 | BARD1 |  |  | 2514 | 0.056 | 0.3432 | No |
| 176 | BICD1 |  |  | 2566 | 0.055 | 0.3382 | No |
| 177 | WEE1 |  |  | 2687 | 0.050 | 0.3249 | No |
| 178 | HSDL2 |  |  | 2688 | 0.050 | 0.3259 | No |
| 179 | ZEB1 |  |  | 2764 | 0.048 | 0.3179 | No |
| 180 | FLCN |  |  | 2765 | 0.048 | 0.3188 | No |
| 181 | SGSM2 |  |  | 2780 | 0.048 | 0.3181 | No |
| 182 | HEG1 |  |  | 2806 | 0.047 | 0.3160 | No |
| 183 | TSPAN3 |  |  | 2828 | 0.046 | 0.3145 | No |
| 184 | ATP1B2 |  |  | 2838 | 0.046 | 0.3143 | No |
| 185 | FGFR2 |  |  | 2844 | 0.046 | 0.3146 | No |
| 186 | SEL1L3 |  |  | 2895 | 0.044 | 0.3095 | No |
| 187 | FAM219A |  |  | 2914 | 0.044 | 0.3082 | No |
| 188 | ABCD3 |  |  | 2974 | 0.042 | 0.3020 | No |
| 189 | TLE4 |  |  | 2975 | 0.042 | 0.3028 | No |
| 190 | NPAS3 |  |  | 2988 | 0.041 | 0.3022 | No |
| 191 | CTNND1 |  |  | 3013 | 0.041 | 0.3001 | No |
| 192 | STX16 |  |  | 3016 | 0.041 | 0.3007 | No |
| 193 | ING3 |  |  | 3019 | 0.041 | 0.3013 | No |
| 194 | SLC35D1 |  |  | 3023 | 0.040 | 0.3017 | No |
| 195 | ABAT |  |  | 3059 | 0.040 | 0.2983 | No |
| 196 | FAM19A5 |  |  | 3130 | 0.038 | 0.2907 | No |
| 197 | INSIG1 |  |  | 3136 | 0.038 | 0.2908 | No |
| 198 | REEP3 |  |  | 3147 | 0.037 | 0.2904 | No |
| 199 | PDGFC |  |  | 3169 | 0.037 | 0.2886 | No |
| 200 | IQSEC1 |  |  | 3221 | 0.035 | 0.2832 | No |
| 201 | FHOD3 |  |  | 3226 | 0.035 | 0.2834 | No |
| 202 | NUMA1 |  |  | 3232 | 0.035 | 0.2835 | No |
| 203 | LARP7 |  |  | 3234 | 0.035 | 0.2841 | No |
| 204 | TLK1 |  |  | 3326 | 0.032 | 0.2739 | No |
| 205 | RGL1 |  |  | 3374 | 0.031 | 0.2689 | No |
| 206 | PDLIM3 |  |  | 3422 | 0.030 | 0.2638 | No |
| 207 | ZFHX4 |  |  | 3437 | 0.029 | 0.2628 | No |
| 208 | IFITM3 |  |  | 3449 | 0.029 | 0.2620 | No |
| 209 | PLCE1 |  |  | 3465 | 0.029 | 0.2608 | No |
| 210 | STXBP3 |  |  | 3477 | 0.029 | 0.2601 | No |
| 211 | SCRN1 |  |  | 3507 | 0.028 | 0.2571 | No |
| 212 | DENND1A |  |  | 3531 | 0.027 | 0.2549 | No |
| 213 | TMEM170A |  |  | 3589 | 0.026 | 0.2486 | No |
| 214 | PUS7 |  |  | 3638 | 0.025 | 0.2434 | No |
| 215 | ATP10D |  |  | 3714 | 0.022 | 0.2349 | No |
| 216 | ARHGAP5 |  |  | 3722 | 0.022 | 0.2345 | No |
| 217 | RAI14 |  |  | 3742 | 0.022 | 0.2326 | No |
| 218 | LSS |  |  | 3790 | 0.020 | 0.2274 | No |
| 219 | SALL1 |  |  | 3800 | 0.020 | 0.2268 | No |
| 220 | CTNNA1 |  |  | 3806 | 0.020 | 0.2266 | No |
| 221 | FERMT2 |  |  | 3823 | 0.020 | 0.2250 | No |
| 222 | NCKAP5 |  |  | 3860 | 0.018 | 0.2211 | No |
| 223 | PTAR1 |  |  | 3879 | 0.018 | 0.2193 | No |
| 224 | FOS |  |  | 3899 | 0.018 | 0.2174 | No |
| 225 | TOB2 |  |  | 3901 | 0.018 | 0.2176 | No |
| 226 | GCA |  |  | 3924 | 0.017 | 0.2153 | No |
| 227 | ZMAT1 |  |  | 3925 | 0.017 | 0.2157 | No |
| 228 | SPATA13 |  |  | 3941 | 0.017 | 0.2142 | No |
| 229 | SPRED1 |  |  | 3989 | 0.016 | 0.2089 | No |
| 230 | SRGAP2C |  |  | 3997 | 0.015 | 0.2084 | No |
| 231 | PDCD4 |  |  | 4013 | 0.015 | 0.2069 | No |
| 232 | HEATR5A |  |  | 4069 | 0.013 | 0.2006 | No |
| 233 | JADE1 |  |  | 4085 | 0.013 | 0.1990 | No |
| 234 | ETV5 |  |  | 4087 | 0.013 | 0.1992 | No |
| 235 | SCHIP1 |  |  | 4109 | 0.012 | 0.1969 | No |
| 236 | EGR1 |  |  | 4134 | 0.012 | 0.1943 | No |
| 237 | SMAD5 |  |  | 4201 | 0.010 | 0.1866 | No |
| 238 | DTNA |  |  | 4203 | 0.009 | 0.1866 | No |
| 239 | REST |  |  | 4235 | 0.009 | 0.1831 | No |
| 240 | PPA1 |  |  | 4255 | 0.008 | 0.1810 | No |
| 241 | SEPT2 |  |  | 4315 | 0.006 | 0.1741 | No |
| 242 | LRIG3 |  |  | 4324 | 0.006 | 0.1732 | No |
| 243 | PDLIM5 |  |  | 4368 | 0.005 | 0.1682 | No |
| 244 | SOX9 |  |  | 4503 | 0.001 | 0.1522 | No |
| 245 | MID1 |  |  | 4556 | -0.000 | 0.1460 | No |
| 246 | ACBD5 |  |  | 4567 | -0.001 | 0.1448 | No |
| 247 | MSI1 |  |  | 4661 | -0.003 | 0.1338 | No |
| 248 | RFX4 |  |  | 4672 | -0.003 | 0.1327 | No |
| 249 | EMC2 |  |  | 4744 | -0.005 | 0.1243 | No |
| 250 | STK17B |  |  | 4780 | -0.006 | 0.1202 | No |
| 251 | GLI3 |  |  | 4782 | -0.006 | 0.1202 | No |
| 252 | PDCD10 |  |  | 4783 | -0.006 | 0.1203 | No |
| 253 | ZDHHC2 |  |  | 4789 | -0.006 | 0.1198 | No |
| 254 | SHROOM3 |  |  | 4902 | -0.008 | 0.1066 | No |
| 255 | IRF2BP2 |  |  | 4927 | -0.009 | 0.1039 | No |
| 256 | LRP4 |  |  | 4954 | -0.010 | 0.1010 | No |
| 257 | NRBP2 |  |  | 4967 | -0.010 | 0.0998 | No |
| 258 | CELSR1 |  |  | 4969 | -0.010 | 0.0999 | No |
| 259 | PHYHIPL |  |  | 4985 | -0.010 | 0.0983 | No |
| 260 | NBPF14 |  |  | 5001 | -0.011 | 0.0967 | No |
| 261 | YAP1 |  |  | 5041 | -0.012 | 0.0923 | No |
| 262 | PPM1K |  |  | 5046 | -0.012 | 0.0921 | No |
| 263 | TAOK2 |  |  | 5207 | -0.016 | 0.0733 | No |
| 264 | OAT |  |  | 5237 | -0.017 | 0.0701 | No |
| 265 | CTDSP2 |  |  | 5249 | -0.017 | 0.0692 | No |
| 266 | GULP1 |  |  | 5255 | -0.017 | 0.0689 | No |
| 267 | TRPS1 |  |  | 5267 | -0.017 | 0.0679 | No |
| 268 | DMD |  |  | 5320 | -0.019 | 0.0621 | No |
| 269 | PREX1 |  |  | 5327 | -0.019 | 0.0618 | No |
| 270 | MGST3 |  |  | 5395 | -0.021 | 0.0542 | No |
| 271 | SEPT3 |  |  | 5444 | -0.022 | 0.0489 | No |
| 272 | DOCK1 |  |  | 5496 | -0.024 | 0.0433 | No |
| 273 | KLF6 |  |  | 5627 | -0.028 | 0.0283 | No |
| 274 | DARS |  |  | 5647 | -0.028 | 0.0266 | No |
| 275 | TBL1X |  |  | 5658 | -0.029 | 0.0260 | No |
| 276 | EFNB2 |  |  | 5685 | -0.029 | 0.0234 | No |
| 277 | TP53 |  |  | 5700 | -0.030 | 0.0224 | No |
| 278 | MOXD1 |  |  | 5709 | -0.030 | 0.0220 | No |
| 279 | GLUD1 |  |  | 5727 | -0.031 | 0.0206 | No |
| 280 | C1orf198 |  |  | 5750 | -0.031 | 0.0186 | No |
| 281 | SEPT9 |  |  | 5792 | -0.033 | 0.0143 | No |
| 282 | EEPD1 |  |  | 5798 | -0.033 | 0.0144 | No |
| 283 | RHOJ |  |  | 5872 | -0.035 | 0.0063 | No |
| 284 | ZCCHC3 |  |  | 5890 | -0.035 | 0.0050 | No |
| 285 | CDCA7L |  |  | 5899 | -0.036 | 0.0048 | No |
| 286 | FAM114A1 |  |  | 5903 | -0.036 | 0.0051 | No |
| 287 | MAT2B |  |  | 5925 | -0.037 | 0.0033 | No |
| 288 | TCF7L2 |  |  | 5935 | -0.037 | 0.0030 | No |
| 289 | AEBP2 |  |  | 6045 | -0.041 | -0.0092 | No |
| 290 | DDAH1 |  |  | 6076 | -0.042 | -0.0120 | No |
| 291 | ALDH6A1 |  |  | 6109 | -0.043 | -0.0149 | No |
| 292 | LHX2 |  |  | 6148 | -0.045 | -0.0186 | No |
| 293 | GATM |  |  | 6161 | -0.045 | -0.0191 | No |
| 294 | EIF2AK2 |  |  | 6220 | -0.047 | -0.0251 | No |
| 295 | CDO1 |  |  | 6251 | -0.048 | -0.0278 | No |
| 296 | STK39 |  |  | 6252 | -0.048 | -0.0268 | No |
| 297 | ZC3HAV1 |  |  | 6254 | -0.049 | -0.0260 | No |
| 298 | ALDH9A1 |  |  | 6304 | -0.050 | -0.0308 | No |
| 299 | TFAP2C |  |  | 6436 | -0.054 | -0.0454 | No |
| 300 | ALDOA |  |  | 6448 | -0.054 | -0.0456 | No |
| 301 | NADK2 |  |  | 6473 | -0.055 | -0.0474 | No |
| 302 | CASC10 |  |  | 6500 | -0.056 | -0.0494 | No |
| 303 | QKI |  |  | 6517 | -0.057 | -0.0502 | No |
| 304 | ANXA5 |  |  | 6522 | -0.057 | -0.0495 | No |
| 305 | TBC1D1 |  |  | 6559 | -0.059 | -0.0527 | No |
| 306 | FDFT1 |  |  | 6637 | -0.063 | -0.0606 | No |
| 307 | EVI5 |  |  | 6690 | -0.065 | -0.0655 | No |
| 308 | BBX |  |  | 6717 | -0.067 | -0.0673 | No |
| 309 | ZFP36L2 |  |  | 6774 | -0.070 | -0.0726 | No |
| 310 | TGIF1 |  |  | 6888 | -0.075 | -0.0846 | No |
| 311 | ZFP36L1 |  |  | 6893 | -0.076 | -0.0836 | No |
| 312 | CHP1 |  |  | 6896 | -0.076 | -0.0824 | No |
| 313 | SPAG16 |  |  | 6910 | -0.076 | -0.0824 | No |
| 314 | PALLD |  |  | 6929 | -0.077 | -0.0830 | No |
| 315 | MAPK1 |  |  | 6941 | -0.078 | -0.0828 | No |
| 316 | CTBP2 |  |  | 6949 | -0.078 | -0.0821 | No |
| 317 | CASP3 |  |  | 6969 | -0.079 | -0.0828 | No |
| 318 | NDUFB5 |  |  | 7052 | -0.084 | -0.0909 | No |
| 319 | BOC |  |  | 7182 | -0.092 | -0.1045 | No |
| 320 | ENO1 |  |  | 7257 | -0.097 | -0.1114 | No |
| 321 | SEPT11 |  |  | 7355 | -0.104 | -0.1209 | No |
| 322 | SDCBP |  |  | 7454 | -0.113 | -0.1304 | No |
| 323 | MYL12A |  |  | 7482 | -0.115 | -0.1313 | No |
| 324 | RGS16 |  |  | 7541 | -0.122 | -0.1359 | No |
| 325 | PHACTR2 |  |  | 7551 | -0.122 | -0.1345 | No |
| 326 | MMAB |  |  | 7557 | -0.123 | -0.1327 | No |
| 327 | RHOQ |  |  | 7632 | -0.131 | -0.1389 | No |
| 328 | PSAT1 |  |  | 7634 | -0.131 | -0.1365 | No |
| 329 | NFE2L2 |  |  | 7651 | -0.133 | -0.1358 | No |
| 330 | CKB |  |  | 7659 | -0.134 | -0.1339 | No |
| 331 | CACYBP |  |  | 7708 | -0.141 | -0.1369 | No |
| 332 | PCGF5 |  |  | 7719 | -0.142 | -0.1353 | No |
| 333 | JUN |  |  | 7746 | -0.146 | -0.1355 | No |
| 334 | AIF1L |  |  | 7768 | -0.148 | -0.1351 | No |
| 335 | CARHSP1 |  |  | 7812 | -0.154 | -0.1372 | No |
| 336 | EEF1D |  |  | 7817 | -0.154 | -0.1346 | No |
| 337 | PPP2CB |  |  | 7824 | -0.155 | -0.1323 | No |
| 338 | GLO1 |  |  | 7844 | -0.157 | -0.1315 | No |
| 339 | CAPN2 |  |  | 7878 | -0.162 | -0.1322 | No |
| 340 | SH3BGRL |  |  | 7984 | -0.182 | -0.1411 | No |
| 341 | LITAF |  |  | 8015 | -0.188 | -0.1410 | No |
| 342 | SPRY2 |  |  | 8110 | -0.205 | -0.1482 | No |
| 343 | FBXO32 |  |  | 8117 | -0.208 | -0.1448 | No |
| 344 | CHMP2A |  |  | 8134 | -0.213 | -0.1425 | No |
| 345 | JUND |  |  | 8150 | -0.214 | -0.1400 | No |
| 346 | REXO2 |  |  | 8200 | -0.229 | -0.1414 | No |
| 347 | ANXA2 |  |  | 8206 | -0.230 | -0.1374 | No |
| 348 | HOPX |  |  | 8211 | -0.231 | -0.1333 | No |
| 349 | CNN3 |  |  | 8228 | -0.234 | -0.1306 | No |
| 350 | PPP1CB |  |  | 8232 | -0.235 | -0.1263 | No |
| 351 | GSTP1 |  |  | 8296 | -0.257 | -0.1288 | No |
| 352 | STK17A |  |  | 8300 | -0.258 | -0.1240 | No |
| 353 | TAGLN2 |  |  | 8313 | -0.261 | -0.1203 | No |
| 354 | GNG12 |  |  | 8323 | -0.264 | -0.1162 | No |
| 355 | PEA15 |  |  | 8347 | -0.272 | -0.1135 | No |
| 356 | PDCD6 |  |  | 8359 | -0.278 | -0.1094 | No |
| 357 | SRI |  |  | 8395 | -0.292 | -0.1078 | No |
| 358 | HMGN3 |  |  | 8452 | -0.316 | -0.1082 | No |
| 359 | MYL6 |  |  | 8495 | -0.339 | -0.1066 | No |
| 360 | LGALS3 |  |  | 8522 | -0.347 | -0.1028 | No |
| 361 | RPS27L |  |  | 8528 | -0.351 | -0.0965 | No |
| 362 | SAT1 |  |  | 8533 | -0.354 | -0.0900 | No |
| 363 | FABP7 |  |  | 8536 | -0.354 | -0.0832 | No |
| 364 | YBX3 |  |  | 8545 | -0.362 | -0.0770 | No |
| 365 | DBI |  |  | 8571 | -0.383 | -0.0724 | No |
| 366 | HSPB1 |  |  | 8572 | -0.383 | -0.0649 | No |
| 367 | GNG5 |  |  | 8575 | -0.384 | -0.0575 | No |
| 368 | GFAP |  |  | 8610 | -0.413 | -0.0534 | No |
| 369 | HIGD1A |  |  | 8616 | -0.419 | -0.0457 | No |
| 370 | MT2A |  |  | 8650 | -0.456 | -0.0407 | No |
| 371 | PHLDA1 |  |  | 8670 | -0.476 | -0.0336 | No |
| 372 | RAB31 |  |  | 8696 | -0.514 | -0.0264 | No |
| 373 | FABP5 |  |  | 8699 | -0.518 | -0.0164 | No |
| 374 | TPI1 |  |  | 8709 | -0.538 | -0.0068 | No |
| 375 | SERF2 |  |  | 8724 | -0.594 | 0.0032 | No |
Table: GSEA details [plain text format]

  

Fig 2: RICHARDS\_NOWAKOWSKI\_SCIENCE\_2017\_VRG\_UPREG: Random ES distribution      
 Gene set null distribution of ES for **RICHARDS\_NOWAKOWSKI\_SCIENCE\_2017\_VRG\_UPREG**
